# Effects of Different Oxidation Methods on the Wetting and Diffusion Characteristics of a High-Alumina Glass Sealant on 304 Stainless Steel

**DOI:** 10.3390/ma17102251

**Published:** 2024-05-10

**Authors:** Changjun Chen, Liwei Sui, Min Zhang

**Affiliations:** 1 Laser Processing Research Center, School of Mechanical and Electric Engineering, Soochow University, Suzhou 215131, China; chenchangjun@suda.edu.cn (C.C.); 13070813383@163.com (L.S.); 2 State Key Laboratory of Advanced Welding and Joining, Harbin Institute of Technology, Harbin 150001, China

**Keywords:** high-alumina glass, 304 stainless steel, pre-oxidation, laser surface melting, wetting

## Abstract

Glass-to-metal seals are a very important element in the construction of vacuum tubes, electric discharge tubes, pressure-tight glass windows in metal cases, and metal or ceramic packages of electronic components. This paper presents the influence of different pretreatment methods on the high-temperature wettability of 304 stainless steel by high-alumina glass sealing. The pretreatment of the steel included laser surface melting and pre-oxidizing. The bonding characteristics of glass and stainless steel directly depend on the wettability in terms of the measured wetting angle, the type of oxide formed at the stainless steel surface, and the microstructural changes during the manufacturing process. The oxide film thickness on the stainless steel surface was evaluated to determine the optimal parameters. The film was wetted with high-alumina glass powder at different temperatures. The results showed that pre-oxidation decreased the wetting angle from 56.2° to 33.6°, while for the laser-melted surface, the wetting angle decreased from 49.8° to 31.5°. Scanning electron microscopy (SEM) revealed that the oxide film on the laser-melted surface was thicker and denser than that formed on the pre-oxidized surface. The present work shows that laser surface melting has a greater beneficial influence on the wetting and diffusion characteristics of 304 stainless steel sealed by high-alumina glass.

## 1. Introduction

Electrical penetration assembly (EPA) is essential electrical equipment in extreme environments and high-consequence applications and is also found in a wide range of applications and industries, such as microsystem technologies, subsea oil drilling, oil and gas components, vacuum ducts, nuclear plants, liquid natural gas (LNG) ships, and liquefied natural gas vessels [1,2]. The safety service of this equipment somewhat depends on the security and reliability of EPAs. Glass-to-metal seals (GTMSs) are broadly adopted in EPAs due to their distinct advantages: robust mechanical strength, high dielectric breakdown strength, and good hermeticity [2]. A typical combination of GMTSs is composed of the following parts: AISI 304/316L and borosilicate glass/high-alumina glass are used as the housing metal and sealing material, respectively [3]. Typically, the GTM compression seals were fabricated using pre-oxidized AISI 304 and borosilicate glass or high-alumina glass with different thermal expansion coefficients (TECs).

High-alumina ultrathin glass is widely used in the electronic information industry owing to its high material strength, high scratch resistance, high toughness, and high wear resistance [4,5], and 304 stainless steel is widely used in the electronics, automobile, aerospace, and manufacturing fields [6,7,8]. Glass-to-304 stainless steel seals are a very important element of the construction of pressure-tight glass windows in metal cases, and metal or ceramic packages of electronic components. Therefore, sealing high-alumina glass and stainless steel is highly important for further application. This process can meet the sealing requirements of mobile phone cover glass and metal shells [9], mobile phone cameras in the mobile phone industry, medical endoscope sealing and window glass sealing in the medical industry, and electrical components in the electronic industry for airtightness and mechanical properties.

To achieve stronger bonding between glass and metal at high temperatures, effective wettability and the diffusion of glass with metal are needed. The molten glass flows over the metal surface and reacts with the oxide layer on the metal surface to achieve stronger bonding [10,11]. To achieve a high-quality glass–metal seal, several requirements must be met; the coefficients of thermal expansion of glass and metal at high temperatures must be similar, and the glass should wet and spread across the metal surface. Achieving the latter requirement depends on the oxide layer thickness and phase constitution at the metal surface [12,13].

During the wetting process, the oxide on the metal surface will react to form a transition layer between the metal and glass. Thus, the properties of glass-to-metal sealing are determined by the thickness, microstructure, and phase constitution of this reaction-product layer. An oxide layer that is too thin will dissolve into the glass and a layer too thick will create a porous structure, both of which will lead to hermetic failure [9,10]. In addition, appropriate types of oxides are needed [14]. Due to the high solubility of CrO and Fe_2_O_3_ in glass, they spread rapidly at the interface, resulting in reduced sealing performance [15].

Although the oxidation of 304 stainless steel (304 SS) has been extensively studied, the main area of concern is oxidation resistance in extreme environments [16,17]. It has been reported that the optimal thickness of oxide films on alloy surfaces is 2–10 μm [13]. In terms of the wettability of glass and stainless steel, Liu et al. [17] found that an oxide film on a metal surface can enhance wettability. In contrast, there are few reports on improving the wettability of stainless steel by laser surface melting. Lawrence and Li [18] worked on this problem to improve the properties of enamel. Chen et al. [15] used a fiber laser to melt the surface of Kovar alloy and found a refined grain size and consequently more grain boundaries. These studies showed that the use of laser radiation on Kovar alloy can achieve the same or better wetting characteristics.

Previous research has mainly treated alloys via a high-temperature pre-oxidation process [19,20,21,22,23,24,25,26,27,28,29], while few scholars have considered the effect of flexible laser treatment on alloys to enhance the wettability between the alloy and glass.

The purpose of this study was to treat the surface of stainless steel by pre-oxidation, laser surface melting, or both. The microstructure and wetting process are revealed to depend on the oxide film formed after different surface treatments. By studying the influence of two conditions on wetting behavior, we provide useful guidance for glass-to-metal sealing.

## 2. Materials and Methods

### 2.1. Material Preparation

The experimental materials used were 304 stainless steel and high-alumina glass. The main chemical composition of 304 stainless steel produced by Taiyuan Iron and Steel is 8.1 wt %, 18.22 wt %, Cr, 0.036 P, 0.002 S, 0.04 C, 0.41 Si, and 1.16 Mn, with a balance of Fe (in wt %). The high-alumina glass used was third-generation Gorilla glass (Gorilla Glass 3) produced by Corning Corporation (Corning, NY, USA) of the United States. The nominal chemical composition of the high-alumina glass is 54.08% SiO_2_, 22.02% Al_2_O_3_, 19.09% Na_2_O, and 4.81% CaO, MgO, and SnO in total (in wt %). The thickness of the glass is 0.5 mm, which is an ultrathin glass.

### 2.2. Pre-Oxidation Treatment

The 304 alloy specimens were abraded through 1200-grade SiC metallographic paper and then ultrasonically cleaned in acetone followed by ethanol. Finally, the specimens were dried. The pre-oxidation treatment of stainless steel was conducted using a box-type resistance furnace (KF1400, Heifei Kejing Furnace Co., Ltd., Hefei, Anhui, China) set at different temperatures and a heating rate of 10 °C/min [21,22,23,24], with the atmosphere during the test being air. The pre-oxidation treatment was carried out at 700 °C, 750 °C, and 800 °C for an isothermal hold time of 20 min by heating the metal specimen. The specimens were then cooled in the furnace to room temperature after pre-oxidation to obtain oxide films of different thicknesses.

There were nine groups of oxidation experiments at each condition, and each group had three parallel specimens. All the specimens were weighed before and after oxidation by a precision balance with an accuracy of 0.1 mg. The oxide thicknesses of the polished cross-sections were measured by a VHX-100K optical digital microscope. The morphology of the surface oxide scales and cross-sections were subsequently examined by scanning electron microscopy (SEM) on a JSM-6480LV instrument, and the phase structure of the oxide was investigated by X-ray diffraction (XRD, Rigaku D/max-A X-ray diffractometer) using CuK^®^ radiation at room temperature. For the experiments, the XRD scan speed was 0.02°/min. The qualitative analysis of oxides on cross-sections was conducted using an electron probe microanalyzer (EPMA, JXA-8100, JEOL, Japan Electronics Co., Ltd., Tokyo, Japan). Importantly, to protect the oxide scales, the oxidized specimens were cladded with copper before cross-section polishing.

### 2.3. Laser Surface Melting Treatment

The 304 stainless steel was cleaned with acetone to avoid contamination. A continuous-wave semiconductor laser with a wavelength of 980 nm for surface melting was melted at 400 W, 450 W, and 500 W with a 2 mm beam diameter and scan speeds of 0.05 m/s, 0.07 m/s, and 0.09 m/s, respectively [30,31]; it was melted under atmosphere without protective gas, allowing the oxygen in the atmosphere to fully react with the stainless steel to obtain oxide films on the melted surface. An overlap between successive laser-melted tracks of approximately 10% was applied to the stainless steel samples. To measure the thickness of the stainless steel oxide film after laser surface melting, the stainless steel samples were sectioned, mounted in a conductive resin, and polished, and then the thickness was measured with a scanning electron microscope. The samples were buried with resin with a function of preserving edge by the casting mounting method. The resin is conductive and it can be observed by SEM directly. The 3D surface morphology of the samples of polished steel and laser-melted surface steel were observed by a confocal microscope (Model VHX-7000, KEYENCE Corporation, Osaka, Japan).

### 2.4. Wetting Assessment

The high-alumina glass was milled in an ethanol medium (ethanol 50 wt % + glass frit 50%) in a rotating mill (QM-3SP4, Nanjing University, China) using ZrO2 containers and ZrO_2_ balls at 450 rpm for 1 h to obtain a glass slurry. The weight ratio of the ball-to-glass frit was 5:1. Some of the glass slurry was dried and sieved through a 200 mesh standard sieve to obtain fine glass powder.

The fine glass powders were ground in an agate mortar and then manually compacted in a mold to a cylinder with a height of ~3 mm and a diameter of ~7.8 mm. The compacted cylinder was placed on a stainless steel substrate and then transferred to a tube furnace (KF1400, Heifei Kejing Furnace Co., Ltd., China). The glass cylinder was then heated from room temperature to the flow temperature at a rate of 6 °C/min in the tube furnace. Three wetting parameters, namely, the apparent contact angle, height, and diameter of the glass cylinder, were recorded using automatic image analysis software. The wetting temperature was set to 1000 °C, 1020 °C, 1050 °C, 1070 °C, and 1090 °C, and the hold time was 30 min. The ratio of the instantaneous height (H) to the initial height (H0) and the ratio of the instantaneous diameter (D) to the initial diameter (D0) were calculated by using the analysis software included in the high-temperature microscope.

The wetted samples were prepared by standard metallographic methods, and the wetting angle was measured through scanning electron microscopy. Microscopy analysis was then carried out to reveal the wetting process and mechanism.

Both the pre-oxidation-treated and laser surface-treated samples, together with the wetted samples, were observed by optical microscopy and SEM analysis. To analyze the type of oxides formed after pre-oxidation and laser surface treatment, pre-oxidized metal specimens and laser surface-treated samples were analyzed using a Bruker Axis Automated Powder X-ray Diffractrometer with Cu Kα (λ = 1.54 A°) X-ray diffraction (XRD) at a scan speed of 0.02°/min, using a voltage of 40 kV and a current of 30 mA. The 2θ scan range was 10–90° with a scan rate of 0.02°/min.

## 3. Results

### 3.1. Surface Oxide Film

**Figure 1:**
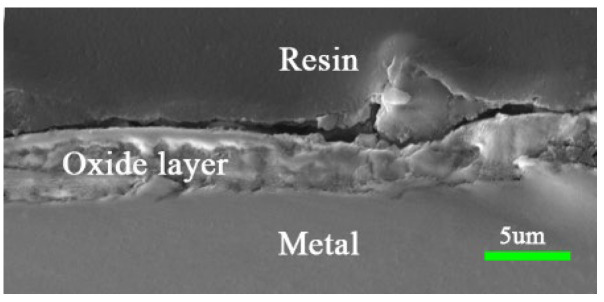
Schematic diagram of the oxide film after high-temperature pre-oxidation (20 min at 750 °C).

Figure 1 shows a representative cross-section of the surface oxide film obtained after holding in the furnace for 20 min at 750 °C. Figure 1 shows that the oxide film closely adhered to the stainless steel, there was no obvious decohesion between them, and the film thickness was evenly distributed, indicating that the oxide film formed under these conditions met the requirements of the subsequent experiments.

**Figure 2:**
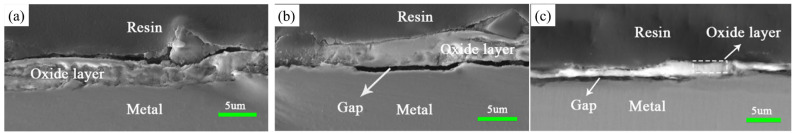
Cross-sectional microscopy of oxide film defects formed during the pre-oxidation process: (**a**) 700 °C; (**b**) 750 °C; (**c**) 800 °C.

However, defects can form in the oxide film obtained by pre-oxidation. For example, when damage occurs, the oxide film thickness distribution is not uniform. As an example, the surface of the oxide film and gaps between the stainless steel substrates are clearly shown in Figure 2. The middle layer of the oxide film also has a gap, which shows that part of the oxide film falls off in the process of grinding or polishing due to the brittle nature of the oxidation film, namely, the oxide film on the stainless steel surface is not strong or dense. Figure 2a shows the gap in the middle part of the oxide film. The gap in Figure 2b appears at the joint with the stainless steel matrix. The gap in Figure 2c also illustrates the joint of the stainless steel matrix and resin. Because high-aluminum glass and stainless steel are dissimilar materials, the surface oxide film plays a role in stability and absorption. Therefore, a stable oxide film forms on the surface of stainless steel during the pre-oxidation process.

To improve the accuracy of the experiment, we measured several groups of samples on average, and the average thickness of the oxide film on the stainless steel surface was measured to be approximately 3 μm.

**Figure 3:**
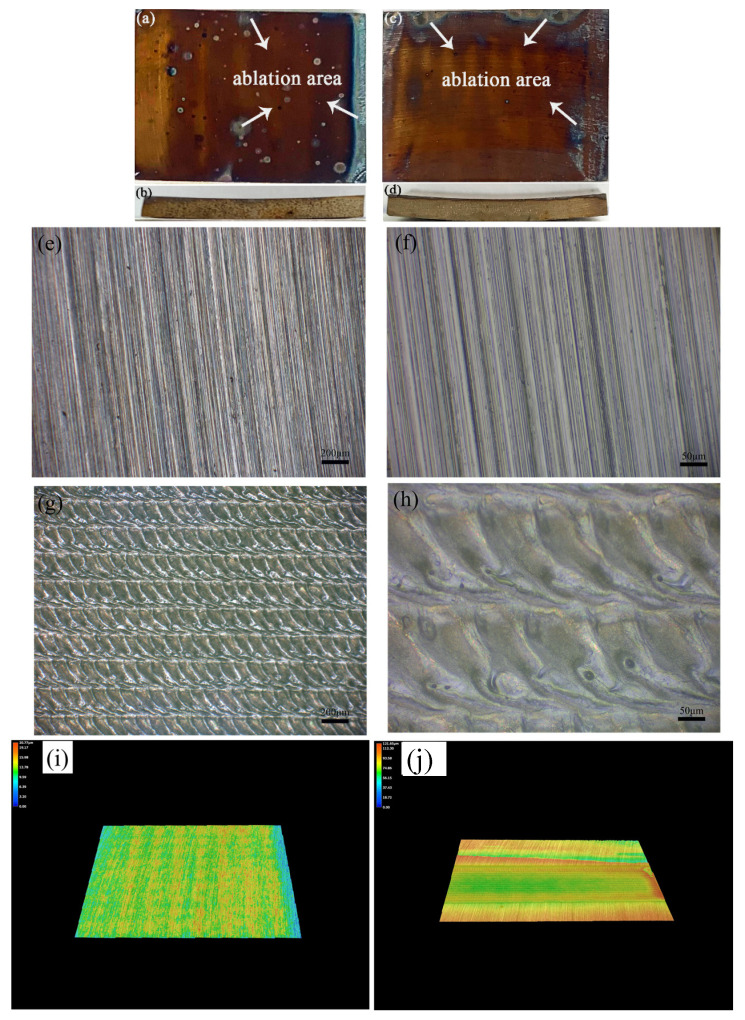
Surface ablation diagram of stainless steel after laser surface melting: (**a**,**b**) 400 W, 0.04 m/s; (**c**,**d**) 550 W, 0.09 m/s; (**a**,**c**) top view; (**b**,**d**) side view; (**e**,**f**) polished steel surface before pre-oxidation treatment and laser surface melting under different magnification; (**g**,**h**) laser-melted steel surface under different magnification; (**i**) 3D surface profile for polished steel surface; (**j**) 3D surface profile for laser-melted steel surface.

To find a suitable way to strengthen the oxide film on the surface of stainless steel, a method using a laser surface melting process was applied to introduce an oxidation film on the stainless steel surface. Figure 3 shows the surface appearance and side view after the laser surface melting process. Figure 3a,c show the appearance of surface 304 stainless steel after laser surface melting, while Figure 3a,c and Figure 3b,d show the top view and side view of 304 stainless steel after the laser surface melting process, respectively.

**Figure 4:**
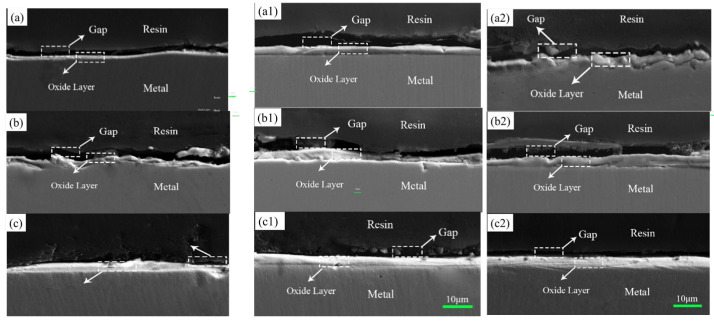
Thickness of the oxide film and the cross-section under different powers and different scanning rates: (**a**) 400 W, 0.05 m/s, 1.9 μm; (**a1**) 400 W, 0.07 m/s, 2.7 μm; (**a2**) 400 W, 0.09 m/s, 3 μm; (**b**) 450 W, 5 mm/s, 3.1 μm; (**b1**) 450 W, 0.07 m/s, 3.2 μm; (**b2**) 450 W, 0.09 m/s, 4.6 μm; (**c**) 500 W, 0.05 m/s, 3.3 μm; (**c1**) 500 W, 0.07 m/s, 3.8 μm; (**c2**) 500 W, 0.09 m/s, 5 μm.

**Figure 5:**
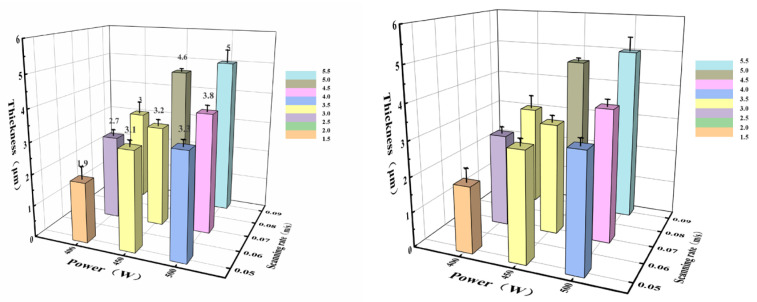
Thickness of the oxide film treated by laser surface melting.

Figure 4 shows the cross-section between the oxide film and the substrate. Figure 5 shows the variation trend of the oxide film thickness under different experimental conditions, i.e., under different laser process parameters. First, we tested the scan velocity under the same experimental conditions, i.e., maintaining constant power. When the scanning rate was maintained at 0.04 m/s, the stainless steel surface was severely ablated and warping deformation occurred. This result suggested that the subsequent sealing work could not be completed well, so the scanning velocity must be changed. The initial scan rate was then set to 0.05 m/s and was gradually increased from 0.05 m/s to 0.07 m/s and 0.09 m/s. When the power was set to 400 W, the obtained surface oxide film thickness changed slightly, and the thicknesses were 1.9 μm, 2.7 μm, and 3 μm. When the power was set to 450 W, the obtained surface oxide film thicknesses were 3.1 μm, 3.4 μm, and 4.6 μm. When the power was set to 500 W, the thicknesses of the obtained stainless steel surface oxide films were 3.3 μm, 3.8 μm, and 5 μm. When the laser power continued to increase to 550 W, the surface of the stainless steel was also seriously ablated, and the bending deformation of the sample was severe, which made it impossible to carry out the next experiment. The surface ablation diagram of the stainless steel is shown in Figure 3. There are straight shallow ridges and they are parallel to each other (see Figure 3e,f). This phenomenon was formed due to the steel surface polished by sandpaper, while the steel surface treated by laser surface melting presented like scales of fish skin (see Figure 3g,h). This fish skin scale surface was formed due to the laser melting in a ceaseless melting process. And Figure 3i,j illustrates the 3D surface profiles by the laser-melted surface sample and the polished steel sample. They illustrate different depth, and the laser surface that was melted had greater depth for laser-melted steel than that of the polished sample.

The experimental results show that the thickness of the oxide film on the surface changed slightly at 400 W under the same power conditions. In addition, the thickness was relatively thin, which did not meet the requirements of further sealing experiments. The thickness of the oxide film at 450 W became thicker than that at 400 W, and the greatest thickness can reach 4.6 μm. The thickness continued to increase at 500 W, reaching 5 μm at 0.09 m/s.

Figure 4 shows that increasing the laser power increased the thickness of the oxide film on the stainless steel surface under the same experimental conditions. Figure 5 shows the variation trend of the oxide film thickness at three power levels in detail. When comparing the thickness of the oxide film under each experimental condition shown in Figure 4 and Figure 5, we finally chose the processing parameters at a speed of 0.09 m/s and a power of 500 W, and the obtained oxide film could also be used for subsequent sealing and wetting experiments.

### 3.2. Oxide Film Interface

**Figure 6:**
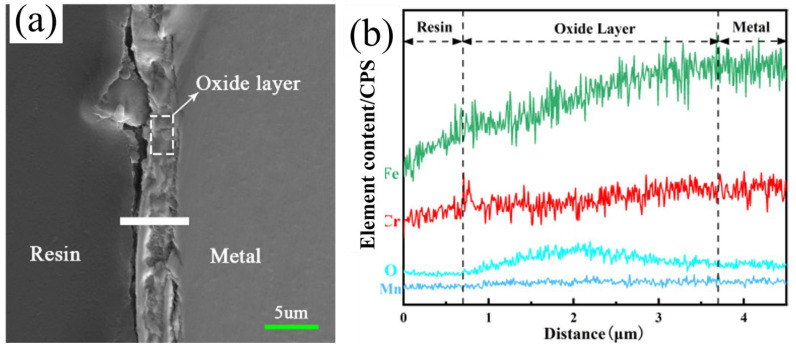
Line scan diagram of the stainless steel oxide layer (pre-oxidation): (**a**) SEM image of the interface; (**b**) O, Fe, and Cr distribution map.

To further explore the chemical composition of the oxide scale on the surface of 304 stainless steel, SEM line scanning analysis was used to analyze the elemental distribution at the interface, and the results are shown in Figure 6 (pre-oxidation process). Similarly, to the experimental conditions of furnace pre-oxidation, O is also obviously enriched at the oxide scale on the surface of the stainless steel, and both Fe and Cr are present in the O-enriched area. This analysis showed that Fe and Cr formed an oxide layer on the stainless steel surface. Iron- and chromium-containing oxides formed on the surface of the stainless steel under both experimental conditions.

O is enriched at the oxide scale on the surface of the stainless steel, and both Fe and Cr are present in the O-enriched area (as shown in Figure 6). Analysis revealed that Fe and Cr formed an oxide layer on the surface of the stainless steel. According to the theory of electrical bonding, when a low-valent oxide is formed on the surface of stainless steel, its electric force line can extend from the large distance formed by the metal cation and its surrounding oxygen ions to ensure bonding with the positive and negative ions in the glass. Now, the glass can form the largest binding force and the smallest repulsive force with the oxide. Therefore, the oxide film formed on the surface of 304 stainless steel by pre-oxidation treatment can improve the wettability of glass and stainless steel.

Comparing Figure 2 and Figure 4, it can be concluded that the oxide film obtained by laser surface treatment has better compactness. The bond between the oxide film and the metal is denser and stronger than the oxide film obtained under the experimental conditions of pre-oxidation. In addition, oxide scale peeling did not occur during the process of grinding and polishing, while peeling easily occurred during the pre-oxidation process.

**Figure 7:**
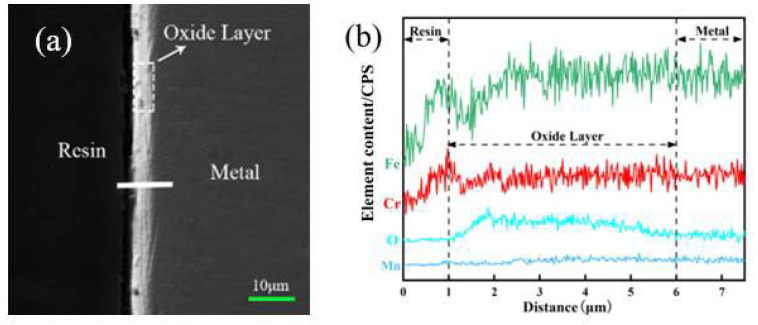
Line scan diagram of the stainless steel oxide layer (laser surface melting): (**a**) SEM image of the interface; (**b**) O, Fe, and Cr distribution map.

Compared with the oxide film treated by pre-oxidation, the final oxide film obtained under laser surface modification is compact and dense, which is beneficial for subsequent wetting or sealing of the glass to the metal (Figure 6 and Figure 7). The literature indicates that the dense oxide film produced on the surface of stainless steel can improve the sealing quality of glass and metal [29]. Compared with that of the pre-oxidized sample, the quality of the oxide film obtained by laser surface treatment is better; the reason might be the better diffusion of oxygen under these conditions [29]. The difference in the diffusivity of oxygen leads to the oxide scale being obtained under the two experimental conditions. The proportions of iron oxide and chromium oxide are different, as is the chemical composition of the iron oxide in the oxide film.

### 3.3. XRD Analysis

**Figure 8:**
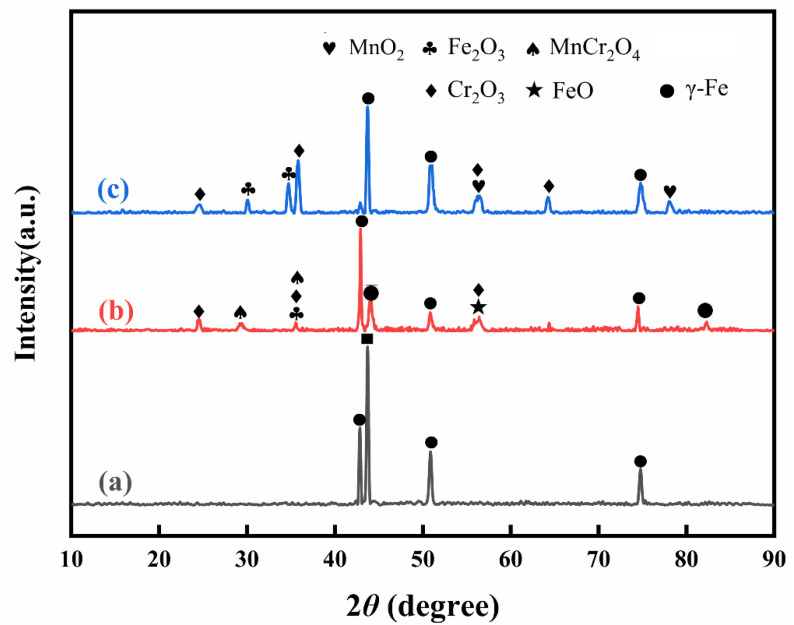
X-ray pattern of the stainless steel surface before and after different oxidation treatments. (**a**) As received; (**b**) high-temperature pre-oxidation; (**c**) laser pretreatment.

X-ray analysis was used to analyze the phases of the stainless steel surface after pre-oxidation in a high-temperature atmosphere furnace and laser surface treatment, and the results are shown in Figure 8. It can be seen from Figure 8 that the surface phase of the stainless steel samples changed significantly after treatment. The surface of the original stainless steel sample mainly contains the γ-Fe phase. After a high-temperature atmosphere furnace pre-oxidation treatment, in addition to the γ-Fe phase, there are also Fe_2_O_3_, FeO, Cr_2_O_3_, MnCr_2_O_4_, and other phases due to the low content of Mn in the stainless steel. In addition, the map obtained by X-ray was consistent with the results obtained by SEM above. After pre-oxidation in a high-temperature atmosphere furnace, oxides containing Fe, Cr, and Mn were generated on the surface of the stainless steel. After the laser surface melting treatment of the stainless steel surface, Fe_2_O_3_, FeO, Cr_2_O_3_, MnO_2_, and other phases were generated, but only the γ-Fe phase was generated because the laser treatment of the stainless steel surface oxide film is thicker than that used for the formation of a high-temperature atmosphere furnace, so the strength of the peak is relatively high. The other strong peaks are mainly γ-Fe, which comes from the stainless steel matrix. Oxides containing Fe, Cr, and Mn were also formed on the surface of the stainless steel after the laser surface melting treatment. Usually, the penetration depth of the X-rays depends on the materials’ type and the X-rays’ type. The penetration depth usually varies from several micrometers to dozens of micrometers. If the oxide scale depth is not thick enough, the XRD results may contain some information of the substrate. In the following sections, it will be revealed that the oxide scale thickness was approximately 10 μm. However, from the XRD results, almost no phases formed on the substrate were not detected. It means that, in the present analysis, the X-rays’ penetration depth is no more than 10 μm for this kind of oxide scale.

When a high-temperature atmosphere furnace is used to perform pre-oxidation, the migration rate of elements and the oxidation rate of metals are accelerated with increasing temperature. According to [32], thermodynamic data can be obtained under constant temperature (550~1000 °C) atmospheric pressure conditions; the lower the required equilibrium partial pressure of oxygen, the more easily the corresponding metal oxidizes, namely, 304 stainless steel Mn and Cr are more likely than Fe to form metal oxides, but the content of two elements in the 304 stainless steel matrix Fe and Cr is much greater than that of Mn. Therefore, on the stainless steel matrix surface, Fe and Cr are more abundant, but the diffusion rate of Mn in Cr_2_O_3_ is two orders of magnitude greater than that of Cr [33]. Therefore, although the diffusion rate of Mn in the stainless steel matrix is much lower than that of Fe, the Cr content is much lower, but there is still MnO_2_ on the surface of the stainless steel. A small amount of MnCr_2_O_4_ spinel was detected on the surface of the stainless steel. Due to the high energy of laser surface treatment, MnO_2_ is generated when Mn diffuses to the surface.

Previous results have shown that Fe^2+^ and Fe^3+^ exist in the oxide, and Fe_3_O_4_ can be regarded as a mixed state of FeO and Fe_2_O_3_ [18,19]. The obtained data are also consistent with those of previous studies [19,20].

### 3.4. Wetting Behavior

**Figure 9:**
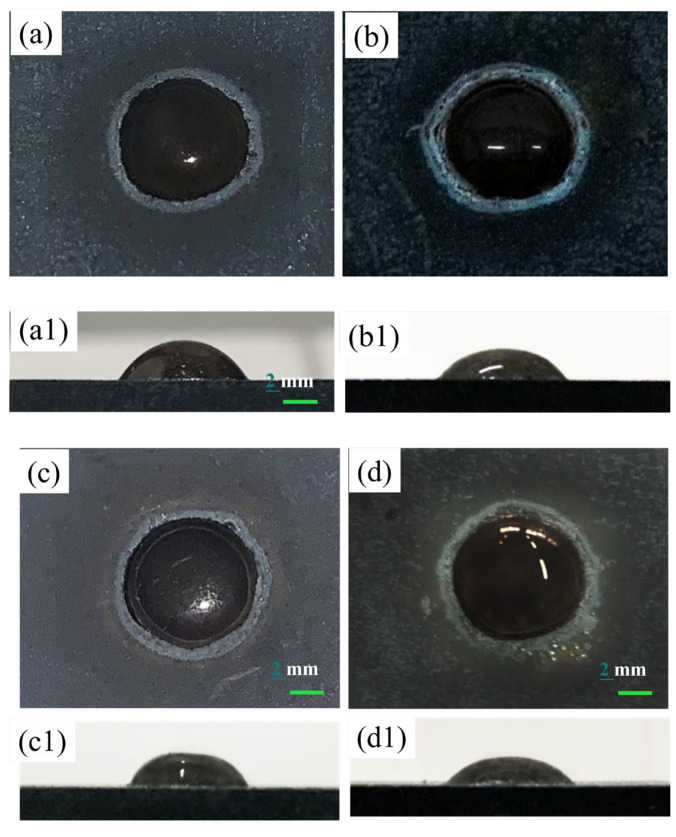

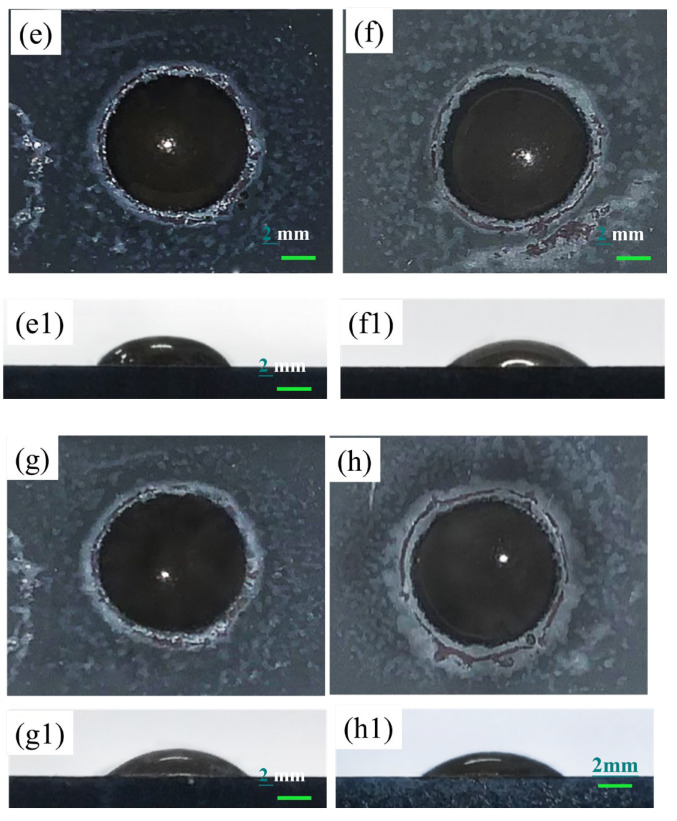
Macrographs of glass on stainless steel after wetting at different temperatures for 30 min at (**a**,**b**) 1020 °C; (**c**,**d**) 1050 °C; (**e**,**f**) 1080 °C; and (**g**,**h**) 1090 °C: (**a**–**h**) top view and (**a1**–**h1**) side view.

**Figure 10:**
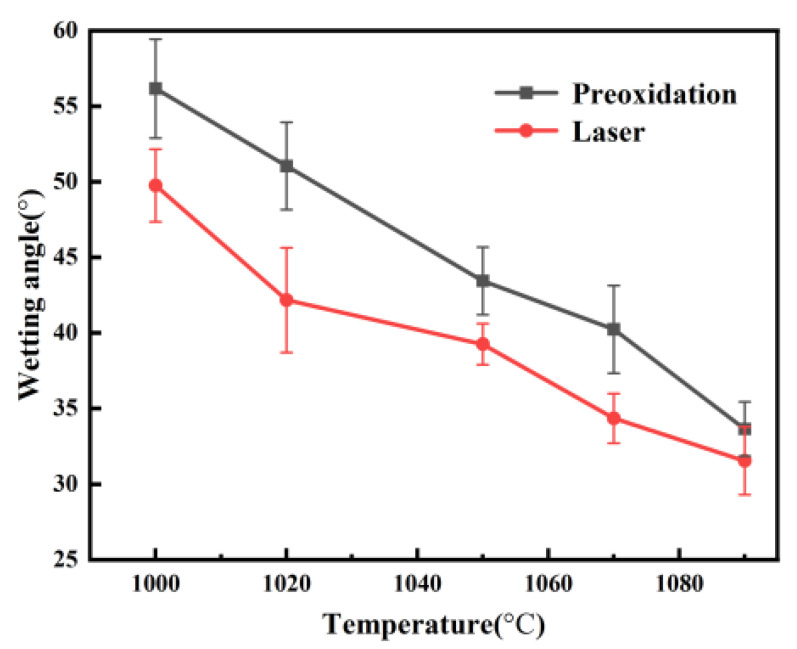
WA as a function of temperature for the wetting of stainless steel by glass for 30 min at different holding temperatures in a furnace for pre-oxidized and pre-laser-treated samples.

In the furnace filled with atmospheric air, the samples with a roughness of 0.156 μm were heated at a rate of 10 K min^−1^ to five different temperatures, namely, 1000 °C, 1020 °C, 1050 °C, 1070 °C, and 1090 °C, held for 30 min, and then cooled in the furnace to room temperature for the purpose of understanding the effect of temperature on the wetting process and finally comparing the effects of the two oxidation methods. From Figure 9, it can be concluded that the variation tendency of the wetting angle is similar whether it is pre-oxidized or laser-treated, and both of these trends continue to decrease the wetting angle. Figure 10 shows the change trend of the wetting angle at different holding temperatures. The contact angle gradually decreases with increasing holding temperature. On the one hand, when the holding temperature increases from 1000 °C to 1090 °C, the wetting angle decreases from 56.16° to 33.65° under the experimental conditions of holding the pre-oxidized sample for 30 min. On the other hand, the wetting angle decreases from 49.76° to 31.54° after laser treatment with a laser power of 500 W. Figure 9 shows that the glass on the surface of the stainless steel is flat in a spherical crown, indicating that wetting performance is enhanced with increasing holding temperature, and a halo is found at the edge of the droplet (see Figure 9). As the temperature increases, the effect of wetting the glass on the stainless steel is enhanced at the same time.

The spread area of the glass on the stainless steel gradually increases with increasing temperature. With increasing distance, the radius of the aperture gradually increases. Figure 9 shows that the larger the aperture formed around the glass droplet, the smaller the wetting angle and the better the wettability. The above phenomenon shows that the formation of a halo depends on the temperature [21]. A further increase in the holding temperature is also beneficial for the generation of the aperture. Therefore, it is believed that the formation of apertures may be a key factor for the good wettability of stainless steel with glass.

Figure 10 shows the wetting angle as a function of the holding temperature for the wetting of stainless steel with glass in atmosphere and pre-oxidized samples heated in a furnace at the same temperature, and the same holding time resulted in poorer wettability than for those pretreated by laser surface melting. The initial wetting angles with a holding time of 30 min were 56.16° and 49.76° for pre-oxidized stainless steel and laser-treated stainless steel, respectively. Lawrence and Li [25] noted that Nd:YAG laser treatment can significantly reduce the wetting angle due to an increase in the polar component of the surface energy. Moreover, an increase in the polar component of the surface energy has a positive effect on the wettability and adhesion of the alloy.

The thermal expansion coefficient of the two materials under high-temperature conditions must be considered when sealing high-alumina glass and stainless steel, and the thermal expansion coefficient must also be close to prevent breakage during the welding process. At the same time, it is essential to consider that the chemical reaction between aluminum glass and the oxide layer on the surface of stainless steel will produce new substances after wetting at high temperatures. The reaction between high-alumina glass and stainless steel is a complex process during wetting. This process involves not only the presence of oxides on the surface of stainless steel but also the presence of metal elements, metal oxides on the surface, and the melting of high-temperature glass, resulting in mutual diffusion between them.

**Figure 11:**
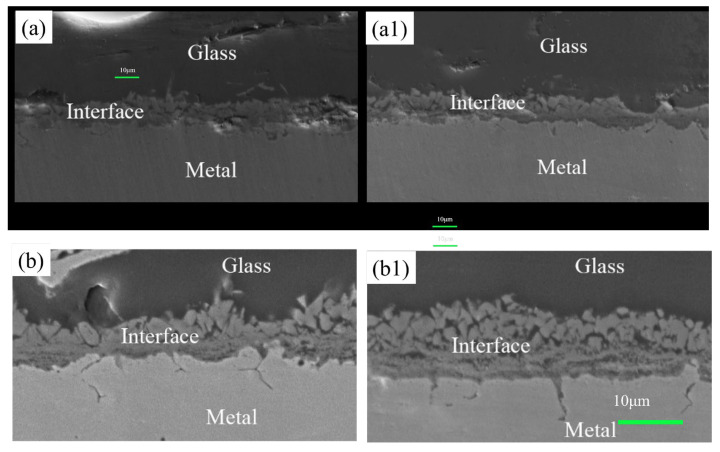

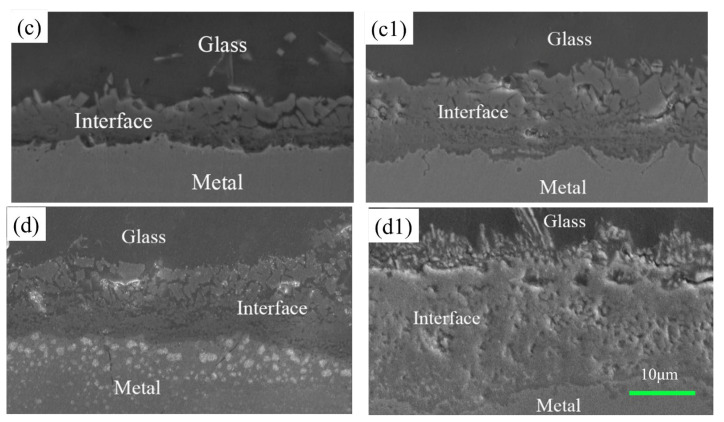
Wetting interface diagrams of pre-oxidized and laser-treated samples at different temperatures: (**a**,**a1**) 1000 °C; (**b**,**b1**) 1020 °C; (**c**,**c1**) 1050 °C; (**d**,**d1**) 1070 °C; (**a**–**d**) pre-oxidation treatment; (**a1**–**d1**) laser surface melting treatment.

To reveal the wetting and diffusion characteristics of glass and stainless steel, wetting samples were mounted, ground, and polished, and they were then observed under a scanning electron microscope to demonstrate the microscopic morphology of the wetting interface between glass and stainless steel. The types and distribution of elements at the interface are shown in Figure 11.

**Figure 12:**
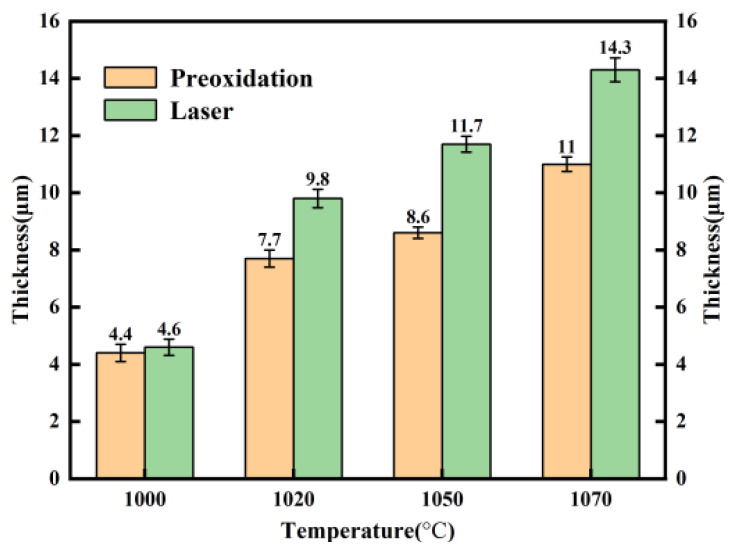
Average thickness of the wetted sample reaction layer at different temperatures.

Figure 11 shows the interface diagram in the boundary region between the high-alumina glass and 304 stainless steel after the wetting process. Figure 11 shows that the reaction layer of the pre-oxidized sample is sparser than that of the laser-treated sample. In addition, the interface of stainless steel has an obvious gap, while the laser-treated sample is denser without a gap. The surface oxide layer of the pre-oxidized samples (a, b, c, d) is relatively sparser. However, after laser surface treatment (a1, b1, c1, d1), the samples exhibit less delamination at the interface, and the interface between glass and stainless steel is continuous and dense. This is due to the formation of a dense oxide layer during laser processing. Figure 12 shows that with increasing temperature, the thickness of the reaction layer gradually increases for both the pre-oxidized sample and the laser-treated sample. This is the result of the diffusion of elements. On the one hand, the stainless steel continues to oxidize; on the other hand, the metal elements react with the elements in the glass to form new compounds. After laser treatment, the thickness of the interface reaction layer is greater than that after pre-oxidation in the high-temperature atmosphere furnace. A thicker reaction layer indicates that the binding strength of glass and stainless steel may increase at the same time, and the wetting effect may also improve.

As for stainless steel (P, where P represents the pre-oxidation sample) under the same wetting conditions, the reaction layer of the pre-oxidized samples is relatively thin, and obvious gaps appear. The reason may be that the oxide layer generated during the pre-oxidation process is not dense enough, and the oxide film can easily spall off, which hinders the continuous diffusion of oxygen and the outward diffusion of iron. The results cause a slower diffusion rate, resulting in the formation of reactive layers with gaps thinner than those in the laser surface samples. In contrast, the oxide scale on the surface of stainless steel (L, where L represents the laser surface-treated sample) is denser, and the metal surface grains are finer after laser treatment than after pre-oxidation treatment. Therefore, the oxygen and iron can easily move inward and outward to form an interfacial reaction layer. Therefore, it can be speculated that the dense surface oxide layer and the refined metal surface provide easier diffusion channels, and the diffusion rate is faster than that of the pre-oxidized sample. This result means that the wetting effect of the samples after laser surface treatment is better than that of the pre-oxidized samples. Figure 12 shows the thickness of the reaction layer after wetting the pre-oxidized samples and the laser surface-treated samples at different temperatures. We can understand this more intuitively.

**Figure 13:**
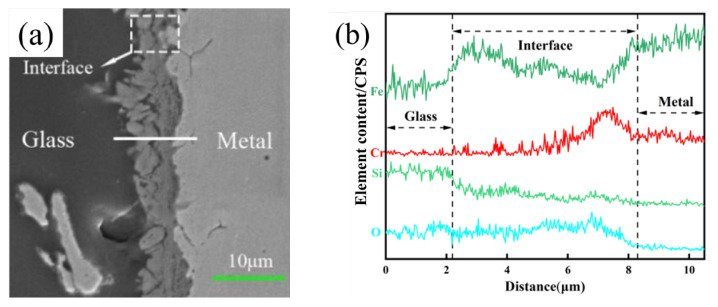
Line scan of the wetting interface (pre-oxidation): (**a**) SEM image of the interface; (**b**) O, Si, Fe, and Cr elemental distribution map (1020 °C).

**Figure 14:**
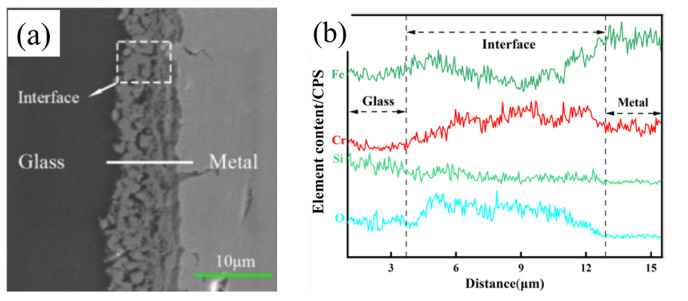
Line scan of the wetting interface (laser treatment): (**a**) SEM image of the interface; (**b**) O, Si, Fe, and Cr distribution map (1020 °C).

To analyze the diffusion behavior at the wetting interface, line scan analysis by SEM was used to analyze the reaction layer, and the results are shown in Figure 13 and Figure 14. The reaction layer of high-alumina glass and stainless steel at high temperatures is followed by the glass area, reaction layer, and metal area. In addition, the scanning route to detect the main elements is from left to right. By observing the element content, the interface can be divided into three parts according to the distribution of the elements: the stainless steel (raw material) area, the interface reaction area, and the glass area. Through the line scan analysis, it can be seen that the various elements change, while the surface scan shows the diffusion of various elements more directly.

**Figure 15:**
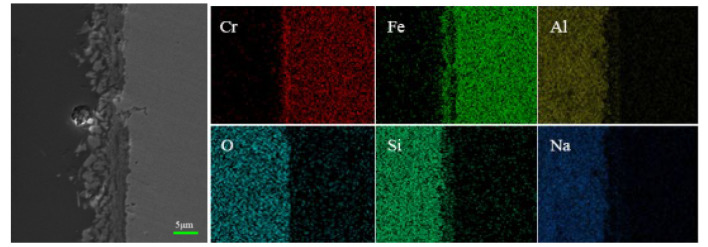
Interface after wetting and its corresponding element distribution (pre-oxidation, 1020 °C).

**Figure 16:**
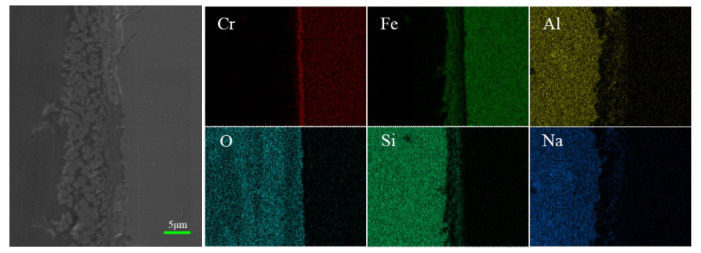
Interface after wetting and its corresponding element distribution (laser treatment, 1020 °C).

Figure 15 and Figure 16 show the wetting interface after the wetting process and its corresponding elemental distribution. First, it can be seen from the generated data that the contents of Si, Al, Na, and O are greater in the glass zone, and in the reaction zone, due to the presence of oxides, the content of O is still high, which diffuses into the reaction zone. There is less Al and Na, so the element content begins to decrease. At this time, Fe is enriched here, indicating that the reaction zone is dominated by iron oxides, Cr rises abruptly near the stainless steel substrate, and Cr is abundant there. For oxides of Cr, the results obtained by the line scan can be verified by the surface scan, and the enriched area of each element is consistent with the line scan. In the stainless steel area, Fe and Cr are the main elements, and the contents of other elements are very small. The results show that Fe_2_SiO_4_ is formed in addition to the oxides of Fe and Cr after wetting, and FeO can react with SiO_2_ to form Fe_2_SiO_4_, indicating that in the process of wetting, in addition to the phenomenon of element diffusion, it is also accompanied by chemical reactions. The results are consistent with the literature.

The oxide layer generated during pretreatment dissolves into the glass, and a further reaction occurs during the wetting process, at which time Fe_2_SiO_4_ is produced. This is reflected in the rapid response zone. The surface of the stainless steel will be refined by a laser. At this point, there will be more diffusion paths within it, and oxygen diffusion will result in a thicker reaction layer. Moreover, FeO can react with SiO_2_ to form Fe_2_SiO_4_, and the excess oxide diffuses into unsaturated glass after the interface reaches saturation [26,27]. The literature indicates that good adherence of glass on alloys can be obtained as a result of the diffusion and saturation of the glass at the interface with FeO [28]. The surface FeO concentration of the sample after laser surface melting treatment is greater than that of the pre-oxidized sample, so it has better wettability.

**Figure 17:**
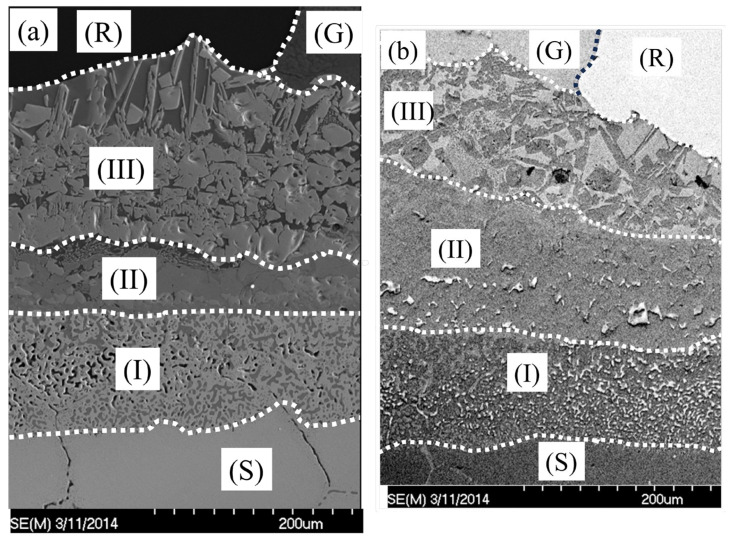
Microstructure of the different zones after wetting for (**a**) 304 steel (P) and (**b**) 304 steel (L) (1100 °C for 100 min) (R indicates the resin zone; S indicates the 304 steel substrate; G represents glass; I indicates porous 304 steel internal oxidation; II indicates the interlayer; and III indicates the mixed zone between the glass and the Fe2SiO4 phase) (P—pre-oxidation, L—laser surface melting).

Figure 17 shows the wettability and diffusion phenomenon for the glass-to-304 stainless steel alloy in the interfacial zone from the 304 steel substrate to the glass with a holding time of 1090 °C. There are five distinct domains:

(a) Original 304 stainless alloy with its own microstructure (indicated as S for the substrate);

(b) Zone I indicates the porous 304 steel zone (as confirmed by the Fe-depleted zone in Figure 8 and Figure 9). This zone is also called the internal oxidation zone. In this zone, the internal oxide scale thickness for 304 steel (laser treatment) is approximately 121.95 µm, which is greater than that for 304 steel (pre-oxidation), which is only 109.76 µm. This zone formed in the original 304 steel substrate.

(c) Zone II indicates the interlayer. This interlayer acts as the transition layer between the substrate alloy and glass. The thickness of 304 steel (L) is greater than that of 304 steel (P).

(d) Zone III shows a mixed zone (confirmed as a mixed zone of glass and Fe_2_SiO_4_). In this area, the phase distribution was more uniform in the 304 steel (L) than in the 304 steel (P).

(e) The glass zone shows the borosilicate glass side.

### 3.5. Mechanism

**Figure 18:**
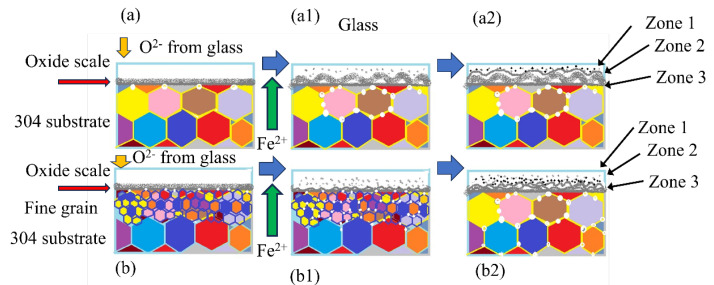
Mechanism of possible formation at the interface for the laser-treated and pre-oxidized samples. (**a**–**a2**): wetting and diffusion process on the pre-oxidation-treated 304 substrate; (**b**–**b2**): wetting and diffusion process on the laser-melted surface 304 substrate. (Zone I: porous Fe-depleted glass; Zone II: FeO-containing interlayer; Zone III: mixed zone; the substrate refers to 304 stainless steel base alloy and the fine grain size refers to the laser-melted zone.)

This result further implies that the steel substrate has a significant influence on the integrity of the oxide scale and the mechanism of the steel/oxide interface. Based on the abovementioned findings, the laser surface-induced grain refinement effect of the steel substrate surface on the adhesive properties was proposed to reveal the interfacial phenomenon and formation mechanism of the tight oxide scale, as schematically shown in Figure 18. A higher concentration gradient of ion diffusion in the coarse-grained steel appears at the grain boundaries, which results in nonuniform oxidation over the entire surface of the steel substrate (Figure 18a). The oxide scale generated on the coarse-grained steel substrate (formed by the pre-oxidation treatment process) thereby leads to nonhomogenous compressive stress. The residual stress can be relieved during the subsequent cooling process, and some spallation failure can be observed on the steel substrate at room temperature (Figure 18(a1,a2)). In comparison with coarse-grained steel, the intergranular oxide scale formed on fine-grained steel can act as a wedge when cooled to room temperature (Figure 18b). As such, a homogenous compressive stress can be dispersed in the uniform oxide scale formed. Previous research reported that hybrid coatings were deposited on a substrate of AISI 321 stainless steel using a combination of plasma-denotation, vacuum-arc, and subsequent high-current electron beam (HCEB) treatment. And electron beam treatment of a hybrid coating surface induced higher adhesion, decreased the intensity of surface wear, and increased corrosion resistance in a sulfuric acid solution [34]. Similar positive results have also been reported by Lobnig [35]. Therefore, in the present study, after laser melting, the modified layer has compact and dense coating. Then, in the following wetting and spreading process, there is no doubt that it exhibited better wetting performance.

The free energy of forming metal oxides of the main elements of Fe, Ni, Cr, and Mn in 304 stainless steel when heated at 700 °C and 1450 °C can be calculated following Ref. [36]. As the melting points for Fe, Ni, Cr, and Mn are 1535 °C, 1453 °C, 1867 °C, and 1244 °C, respectively [33], we assume that the surface temperature is 1450 °C for the following discussion. For pure iron, there are three different oxides, i.e., wustite (FeO), magnetite (Fe_3_O_4_), and hematite (Fe_2_O_3_). Among the Fe, Ni, Cr, and Mn in 304 stainless steel alloys, Mn requires the lowest free energy for oxidation at 700 °C and Cr requires the lowest free energy at 1450 °C. The Cr and Fe sequences at 700 °C and the Mn and Fe sequences at 1460 °C are listed in Table 1. Thus, according to Ref. [36], for 304 stainless steel, ${MnO}_{2}$, FeO, and ${Cr}_{2}O_{3}$ are easily oxidized at 700 °C and 1450 °C.

## 4. Conclusions

In the present study, the surface of stainless steel was treated by two kinds of oxidation methods: furnace pre-oxidation and laser surface treatment. Glass wetting on the steel was conducted on both treated samples to observe the wetting and interfacial reactions. The main conclusions are listed in the following description:

(1) The thickness of the oxide layer on the surface of the pre-oxidized sample increased with increasing temperature; it reached 3 μm at 750 °C with a holding time of 20 min. The oxide layer thickness increased with increasing power and scanning rate; it reached 5 μm in thickness at 500 W after a laser scanning velocity of 0.09 m/s. The quality of the oxide film after laser treatment was denser and more compact than that after pre-oxidation. A denser oxide film can bond tightly with stainless steel without the gap or oxide film peeling-off phenomenon.

(2) In the present study, pre-oxidation treatment decreased the wetting angle from an initial value of 56.16° to a final value of 33.65°, while the wetting angle of the laser-melted surface decreased from 49.76° to 31.54°. An increase in temperature will increase the wettability and decrease the wetting angle. The wetting angle of the sample treated by the laser was smaller than that of the sample treated by pre-oxidation at the same wetting temperature and holding time.

(3) The surface grain size of stainless steel will be refined, and more grain boundaries will be produced after laser treatment; this can promote diffusion, resulting in a thicker oxidation layer and interface reaction layer in the laser-treated sample. The oxide film on the surface of the pre-oxidized sample easily falls off, which hinders the diffusion of elements. Therefore, there are more gaps in the reaction layer during wetting than in the sample treated by laser surface melting. The results of this work show that better wetting characteristics can be obtained using laser surface treatment under the same wetting conditions.

## Figures and Tables

**Table 1:** Oxidation reaction of 304 stainless steel alloy elements at 700 °C and 1450 °C and the free energy required [37].

Reaction Equation	Free Energy (Kcal (mol O_2_)^−1^)	
	700 °C	1450 °C
$2\mathrm{Fe}+O_{2}\to 2FeO$	−94	−75
$\frac{4}{3}\mathrm{Fe}+O_{2}\to \frac{2}{3}Fe_{2}O_{3}$	−84	−45
$2\mathrm{Ni}+O_{2}\to 2\mathrm{NiO}$	−76	−46
$\mathrm{Mn}+O_{2}\to {MnO}_{2}$	−121	−81
$2Cr+\frac{3}{2}O_{2}\to {Cr}_{2}O_{3}$	−117	−91

Note: The melting points for Fe, Ni, Cr, and Mn are 1535 °C, 1453 °C, 1867 °C, and 1244 °C, respectively.

## Data Availability

No data, models, or code were generated or used during the study.
